# Patient and System Barriers to Early Diagnosis of Oral Cancer in the UK

**DOI:** 10.1111/odi.70125

**Published:** 2025-11-15

**Authors:** Emmanuel Kwateng Drokow, Michael Drinnan, Cecilia Amponsem‐Boateng, Fatemeh Vida Zohoori, Janet A. Wilson, Kamini Shah, Francisca Arboh

**Affiliations:** ^1^ School of Health and Life Sciences Teesside University Middlesbrough UK; ^2^ National Horizons Centre Teesside University Darlington UK; ^3^ Adventist Health Study, Research Affairs Loma Linda University Linda California USA; ^4^ Population and Health Sciences Institute Newcastle University Newcastle upon Tyne UK; ^5^ NHS England‐ North East and Yorkshire North East and Yorkshire UK; ^6^ Teesside International Business School Teesside University Middlesbrough UK

**Keywords:** dentist, diagnostic delay, GP, health‐seeking behaviour, oral cancer

## Abstract

**Objective:**

Oral cancer poses a significant public health challenge worldwide, especially in the UK, where delayed diagnosis negatively impacts patient outcomes and survival rates. This study aims to systematically review and synthesise evidence on patient and system barriers to early diagnosis of oral cancer within the UK context.

**Methods:**

Following PRISMA guidelines, we conducted a systematic review and meta‐analysis of peer‐reviewed studies published between 2000 and 2024. The databases we searched included PubMed, Scopus, EMBASE, Google Scholar and Web of Science including grey literature. Studies examining factors influencing patient and healthcare provider delays in oral cancer diagnosis were included. Key data such as the percentage of referrals by GPs and dentists, delay time, authors' names and year were extracted, and a meta‐analytic approach was used to quantify the impact of barriers and facilitators on diagnostic timelines. The risk of bias was assessed using the Methodological Index for Non‐Randomised Studies (MINORS) tool, and the findings were visualised using Robvis. R studio software was used for the quantitative analysis.

**Results:**

The main patient‐level barriers are psychosocial factors, cost, anxiety, and structure of primary dental care, and healthcare‐level barriers are lack of time and inadequate remuneration. The pooled referral proportion for GPs was 0.49, with a 95% CI of 0.40 to 0.59, derived using a random‐effects model. The pooled proportion for dentists was 0.38, with a 95% confidence interval (CI) of 0.31 to 0.46. A pooled relative risk (RR) of 1.27 (95% CI: 0.96–1.68) was observed when comparing referrals made by general practitioners (GPs) to those made by dentists.

**Conclusion:**

The pooled relative risk indicates a marginally increased probability of referrals by general practitioners compared to dentists; however, the overlapping confidence intervals necessitate a cooperative strategy to enhance referral routes. Addressing these obstacles via specific interventions and policy reform could significantly improve the UK's early detection rates and patient outcomes for oral cancer.

## 1 Introduction

With over 370,000 new cases and 177,000 deaths reported annually, oral cancer is a significant global public health concern (Bray et al. 2018). Improving outcomes for patients includes early detection and enabling timely intervention, which leads to decreased treatment morbidity and an improved survival rate (Warnakulasuriya 2009). Timely detection yields an approximately 90% survival rate, whereas a delayed diagnosis results in a 50% survival rate (BDA 2023; Cancer Research UK 2024). In this context, the British Dental Association (BDA) emphasises that persistent access issues will be a matter of life and death for certain patients.

Recent statistics indicate that there were 9860 incidences of oral cancer in the UK during 2020/21, representing a 12% increase compared to the preceding year (BDA 2023). The disease resulted in over 3000 fatalities in 2021, representing a 46% increase from 2075 a decade earlier (BDA 2023). Although ‘high‐high‐risk’ people, such as older smokers and frequent drinkers, can be identified, there is a notable increase in cases attributed to Human papillomavirus (HPV), which predominantly affects younger individuals who typically do not smoke and consume minimal or no alcohol (Gupta et al. 2016). Oral cancer currently results in a higher mortality rate than road traffic accidents across all UK nations (BDA 2023). Disparities in diagnosis, influenced by geographical location, ethnicity and socioeconomic status, make it more difficult to attain equitable health outcomes.

Comprehending diagnostic delays and health‐seeking behaviour (HSB) is essential for oral cancer patient outcome improvement, especially in the UK, where late‐stage diagnoses and increasing incidence rates persist to challenge public health initiatives and efforts. The promptness of diagnosis is influenced by HSB and is significantly linked to survival rates and therapeutic effectiveness (de Amorim Póvoa et al. 2025). Early interventions made possible by timely diagnosis can decrease the financial burden of late‐stage treatment and related medical expenses (Dwivedi et al. 2023). Nevertheless, considerable diagnostic delays still occur in the UK healthcare system, aggravating healthcare inequities across all demographic groups, even after implementing the referral guidelines and awareness campaigns (Anderson et al. 2021).

Previous literature has cited cultural stigma, low symptom awareness, and fear among patient‐related barriers and systemic barriers such as inadequate accessibility to specialist care and inefficiencies in referral pathways (Scott and Hoskin 2024). Nonetheless, there exists a paucity of research that thoroughly integrates these factors to yield meaningful insights and actions for the UK context. Most current assessments concentrate on general cancer categories or broader geographical locations, resulting in a gap in comprehending the specific issues encountered by oral cancer patients in the UK.

To address this gap, the primary objective of the study was to identify the barriers and facilitators influencing diagnostic delays in oral cancer in the UK. The objective of this study is to explore key patient‐related and system‐level barriers to the early diagnosis of oral cancer. Taking this goal into account, the research will inform healthcare policy and help lessen the impact of oral cancer in the United Kingdom.

## 2 Methods and Materials

### 2.1 Study Design and Protocol Registration

To guarantee a transparent and thorough methodology, this study complied with the Preferred Reporting Items for Systematic Reviews and Meta‐Analyses (PRISMA) standards (Moher et al. 2009; Page et al. 2021). The PRISMA framework improves the reproducibility and reliability of the results by offering a consistent method for reporting, evaluating, and finding pertinent studies. The study protocol was registered with the International Prospective Register of Systematic Reviews (PROSPERO) before the review commenced (CRD42024621523).

### 2.2 Searching Strategy and Data Sources

An extensive search was carried out across five main electronic databases, Scopus, PubMed, Web of Science, EMBASE and Google Scholar as well as grey literature sources, to ensure thorough coverage of the relevant literature. These databases were chosen because they extensively index peer‐reviewed articles on social science, medical and healthcare subjects. Studies published between January 2000 and December 2024 were included in the search to get up‐to‐date information on oral cancer diagnostic delays. The cut‐off date of January 2000 was chosen to ensure that the included studies reflect modern healthcare systems, diagnostic advancements and relevant patient behaviours. Furthermore, studies before 2000 often have inconsistent methodologies and outdated healthcare contexts, limiting their comparability to recent findings.

### 2.3 Keywords and Search Terms

Medical Subject Headings (MeSH) and free‐text terms were used to create a search technique specific to the study's objectives. Keywords consisted of the following: “Oral cancer” AND “mouth cancer” AND “oral cavity cancer,” “Health‐seeking behaviours” AND “help‐seeking behaviours” AND “patient delay,” “Diagnostic delay” AND “diagnosis timing,” “Barriers” AND “facilitators,” “United Kingdom” AND “UK”. (Table S1) These phrases were combined using Boolean operators (OR/AND), and word variations were included using truncation. For example, the singular and plural variants of the phrase “cancer” were captured.

### 2.4 Inclusion Criteria

We included observational studies that explored barriers or facilitators to early oral cancer diagnosis in the UK. These could be quantitative (e.g., surveys, cohort studies), qualitative (e.g., interviews, focus groups), or mixed‐methods in design. The eligibility criteria were framed using the SPIDER tool, which is more appropriate for synthesizing qualitative, quantitative, and mixed‐method observational evidence. The SPIDER components were defined as follows:

S (Sample): Adults diagnosed with or at risk of oral cancer in the UK, as well as healthcare professionals involved in their diagnosis (e.g., GPs, dentists).

PI (Phenomenon of Interest): Barriers and facilitators to timely oral cancer diagnosis.

D (Design): Observational studies, including interviews, surveys, focus groups, cohort studies, or mixed‐methods.

E (Evaluation): Factors influencing diagnostic delay, including patient knowledge, provider practices, referral pathways and system‐related issues.

R (Research type): Qualitative, quantitative, or mixed‐method research.

This inclusive approach allowed us to incorporate diverse sources of evidence, reflecting the multifactorial and interdisciplinary nature of barriers to early oral cancer diagnosis in the UK.

### 2.5 Exclusion Criteria

For this study, we excluded reviews, interventional studies, commentaries and editorials, as these do not provide original data suitable for meta‐analysis. Additionally, we excluded non‐English language publications. While we acknowledge the potential benefits of including studies published in other languages, we restricted our review to English‐language studies due to resource limitations and concerns about the accuracy of AI‐based translation tools, particularly for complex medical and qualitative data. Moreover, given our specific focus on the UK healthcare context, most relevant studies were available in English. Finally, we excluded articles with incomplete data or an insufficient focus on diagnostic delays, as these did not meet the objectives of our systematic review.

### 2.6 Screening Procedure

Duplicate articles were eliminated once all retrieved articles were entered into the Covidence software. Two reviewers (E.K.D and C.A.B) separately examined the abstracts and titles to ensure they were pertinent. Discussions or consultations with a third reviewer (F.A) were used to settle discrepancies during screening. The exclusion and inclusion criteria were used to evaluate the full texts of possibly eligible articles. The PRISMA flow diagram documented the search and selection procedure (Moher et al. 2009).

### 2.7 Data Extraction

A standardised process for data extraction was implemented to guarantee accuracy and consistency in the eligible studies. A predesigned data extraction form was used to extract relevant variables based on the study's objectives and full‐text eligibility. The extraction of the data was carried out by two independent reviewers (E.K.D and C.A.B), and discrepancies were resolved through discussion with the third reviewer (F.A). The extracted variables were the author's name, study design, publication year, sample size, and factors influencing diagnostic delays, including patient level (e.g., fear, awareness) and system level (e.g., referral pathways, provider communication) barriers. In addition to stage and delay data, percentages of medical and dental referrals were retrieved. All eligible study designs were included to ensure a comprehensive synthesis of both quantitative data and qualitative insights, which are essential for understanding complex behavioural and systemic factors.

### 2.8 Quality Evaluation

To assess the quality and methodological riguor of the included studies, we employed the Methodological Index for Non‐Randomised Studies (MINORS) framework complemented by the Robvis tool for visualisation of risk‐of‐bias assessments (McGuinness and Higgins 2021; Slim et al. 2003; Sterne et al. 2016). The MINORS framework was used to systematically evaluate the methodological quality of non‐randomised studies. Each study was assessed based on 12 predefined criteria, with eight items for non‐comparative studies and four additional for comparative studies. Each criterion was scored from 0 to 2 (“0: Not reported, 1: Inadequately reported, 2: Adequately reported”). The total MINORS score for each study was calculated, with higher scores indicating greater methodological robustness. Studies were categorised as follows: Low Quality: Total score of 0–8, Moderate Quality: Total score of 9–16, High Quality: Total score of 17–24.

To align the MINORS evaluation with the established risk‐of‐bias framework, individual criteria were mapped to relevant domains (“Confounding Bias: Baseline equivalence of groups”, “Selection Bias: Inclusion of consecutive patients, Performance Bias: Prospective data collection, Detection Bias: Unbiased assessment of endpoints, Attrition Bias: Loss to follow‐up, Reporting Bias: Endpoints appropriate to study aims”) (Slim et al. 2003). Each criterion score was translated into a risk level: (High Risk: Score = 0, Moderate Risk: Score = 1, Low Risk: Score = 2). Visualisation of these assessments was performed using the Robvis tool, which provided traffic light plots and summary bar charts illustrating the distribution of risk levels across studies.

### 2.9 Missing Information

Selected authors were emailed in an attempt to obtain unclear, missing, or both information. This was not feasible in some cases since the authors did not reply or the necessary outcome had not been assessed by the primary authors. As a result, we analysed each outcome using only the readily accessible data.

### 2.10 Data Analysis

RStudio (version 2024.09.1 + 394) was used to perform data analysis and synthesis. Forest plots were created to measure the relative risk of GP and dentist referrals. Some studies underwent meta‐analysis for stage data. The remaining results were presented in narratives and tables. Narratives and tables were used to report the delay data. Relative Risk (RR) was calculated based on the raw counts for studies reporting frequencies or proportions.

### 2.11 Heterogeneity Assessment/Model Selection

Heterogeneity was assessed using Cochran's *Q* test and *I* ^2^ statistics. Cochran's *Q* is a chi‐square test that evaluates the null hypothesis of homogeneity across studies. Fixed‐effect and random‐effects models were considered to account for the variability between studies regarding population, study design, and outcome measures. The fixed‐effect model assumes that all studies estimate the same underlying effect, making it appropriate when highly homogeneous studies are selected if *I* ^2^ < 60%. The random‐effects model was preferred when there was significant heterogeneity between studies; thus, if *I* ^2^ > 60%. This model accounts for both within and between‐study variance, offering a more generalisable estimate when studies are more diverse. The random‐effects model was employed in cases where heterogeneity is substantial or expected due to variations in study populations, settings, and methodologies.

### 2.12 Publication Bias

Publication bias was assessed through funnel plots and Egger's test (Egger et al. 1997). An asymmetrical funnel plot will suggest an absence of bias, while asymmetry may indicate the presence of publication bias, particularly for smaller studies. Egger's test quantitatively evaluates the degree of asymmetry in funnel plots.

### 2.13 Sensitivity Analysis

Sensitivity analyses were conducted by excluding studies with a high risk of bias or extreme effect sizes to test the robustness of the results (Jpt 2008). This step helps to determine if the findings depended on a small subset of studies or were consistent across the included literature. These methods ensure a comprehensive and methodologically sound data synthesis, facilitating the drawing of reliable conclusions regarding the factors influencing diagnostic delays in oral cancer.

### 2.14 Qualitative Synthesis

Thomas and Harden's thematic synthesis was used to analyse qualitative research (Thomas and Harden 2008). The process of thematic synthesis entails coding each result line by line, grouping the codes into themes, and then conducting additional interpretation to produce analytical themes that provide a fresh perspective on the study's findings. Two reviewers worked independently on this. We reported the synthesis of qualitative research by the ENTREQ principles.

## 3 Results

### 3.1 Searching Data Source Outcome

**FIGURE 1:**
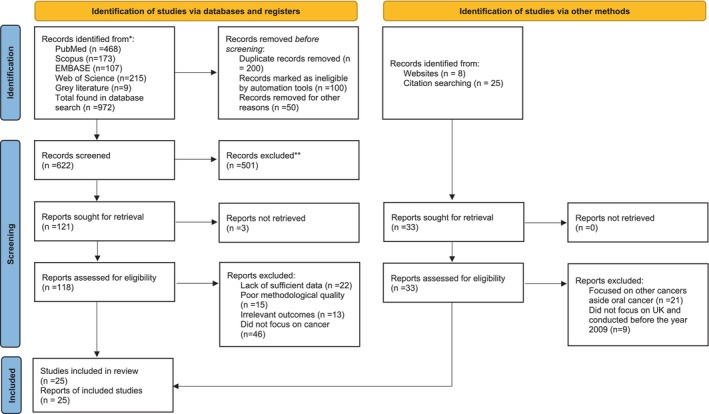
Prisma diagram showing the study process and selection.

Following de‐duplication, 622 articles were retained from the 972 entries identified in the first search of the four primary databases, along with the grey literature search. After filtering titles and abstracts, 501 articles were excluded, resulting in the retrieval of 121 papers. Following the exclusion of 3 articles due to a lack of response from primary authors on clarity and missing data, 118 articles required a comprehensive full‐text eligibility assessment. After assessing 118 full‐text publications against the inclusion criteria, 25 papers were incorporated into the final quantitative and qualitative analysis. Of the 96 papers excluded at the full‐text eligibility stage, 15 were disqualified due to inadequate methodological quality, 22 for insufficient data, 13 for irrelevant outcomes, and 46 for not addressing cancer (Figure 1) (Ahmad et al. 2018; Aminu 2019; Boneham et al. 1997; Borreani et al. 2010, 2008; Crossman et al. 2016; Croucher and Sohanpal 2006; Curtis et al. 2021; Daniel and Rogers 2022; Dwivedi et al. 2023; Dyer and Robinson 2006a, 2006b; Fiske 2000; Grant et al. 2010; Grimes et al. 2017; Hollows et al. 2000; Longhurst 2002; McLeod et al. 2005; Patel et al. 2021; Piggott 2015; Rogers et al. 2007, 2011; Scott et al. 2005, 2009; Singh and Warnakulasuriya 2006; Sweeney et al. 2007; White et al. 2009).

### 3.2 Selected Studies Characteristics

All included studies were observational in design, employing either quantitative, qualitative, or mixed‐methods approaches. The review included 14 quantitative studies, predominantly retrospective cohort studies along with cross‐sectional surveys and prospective observational studies, focusing on patient demographics, referral patterns, and diagnostic delays. Five qualitative studies employed semi‐structured interviews, focus groups, and thematic analyses to explore patient experiences and healthcare provider perspectives. Six mixed‐methods studies combined quantitative data, such as surveys, with qualitative approaches like interviews and thematic synthesis, offering comprehensive insights into barriers to early diagnosis. The included studies collectively covered a wide range of participants, including general practitioners, dentists, and patients, with sample sizes ranging from 20 to 1079 participants. Geographically, the studies spanned various regions of the UK, including London, the Southwest, Southeast, Northeast, and Northwest, as well as Ireland, Wales, and Scotland.

### 3.3 Qualitative Analysis

The synthesis of qualitative findings was based on author‐reported themes extracted from the included qualitative and mixed‐methods studies. We systematically organised these reported themes into two overarching domains: patient‐level barriers and healthcare provider barriers. Patient‐level barriers included factors such as fear, cost concerns, cultural beliefs, and lack of awareness, while healthcare provider‐level barriers encompassed workforce shortages, appointment delays, and inadequate remuneration. This categorisation allowed us to present a structured narrative synthesis that highlights common patterns and key insights across the studies, providing a comprehensive understanding of the multifactorial barriers to early oral cancer diagnosis.

Patient‐Level Barriers:

1. Structure of primary dental care
  - Lack of familiarity with dental systems among recent immigrants
  - Confusion about NHS vs private dental care
  - Limited appointment availability, long treatment plans
2. Cost barriers
  - Dental care perceived as expensive
  - Unclear treatment costs causing anxiety
  - Competing family priorities reduce dental attendance
3. Anxiety (psychosocial factors)
  - Dental fear from past negative experiences
  - Discomfort with treatment providers of the opposite gender
  - Dental anxiety leading to avoidance
4. Lack of support
  - Isolation or lack of caregivers (especially in older adults and care homes)
  - Communication barriers among dementia patients
5. Cultural factors
  - Traditional beliefs about self‐care or family authority over treatment
  - Low knowledge of modern dental practices
6. Lack of awareness
  - Limited understanding of oral cancer signs and risk factors
  - Symptoms mistaken for normal ageing
  - Motivated to seek care only when pain or severe symptoms appeared

Healthcare Provider Barriers:

1. Workforce challenges
  - Insufficient staffing and inconsistent NHS coverage
  - Equipment shortages
2. Time constraints
  - High patient loads limiting examination time
  - Limited time for patient education or discussion
3. Inadequate remuneration
  - Low compensation discourages thorough assessment
  - Underinvestment in modern diagnostic tools
4. Concern over patient compliance
  - Missed appointments and poor follow‐up
  - Limited adherence to screening recommendations
5. Referral system issues
  - Complex pathways delaying specialist referrals
  - Administrative burdens
  - Lack of confidence in biopsy or advanced diagnostics

These summarised barriers reflect a complex interplay of patient beliefs, socioeconomic factors, and structural challenges within healthcare systems, all contributing to diagnostic delays in oral cancer. This thematic structure provided clarity in presenting the multifaceted barriers identified, enabling a cohesive integration of patient and healthcare providers' perspectives. To complement these insights, our quantitative analysis further examined the referral pathways, diagnostic delays, and the distribution of cases identified by various healthcare professionals.

### 3.4 Meta‐Analysis (Quantitative)

Across the included studies, there was no consistent or standardised definition of the terms *“referral pathway”* or *“event.”* For the purpose of this review, we operationally defined a referral pathway as the process by which a patient with potential signs of oral cancer was referred from primary care (either a general practitioner or a dentist) to specialist services for further assessment. An event was considered to represent a single recorded referral for a suspected case of oral cancer, as documented in the primary studies. This working definition allowed for consistency in data interpretation across the heterogeneous reporting styles of the included research.

### 3.5 Pooled GP and Dentist Referrals

**FIGURE 2:**
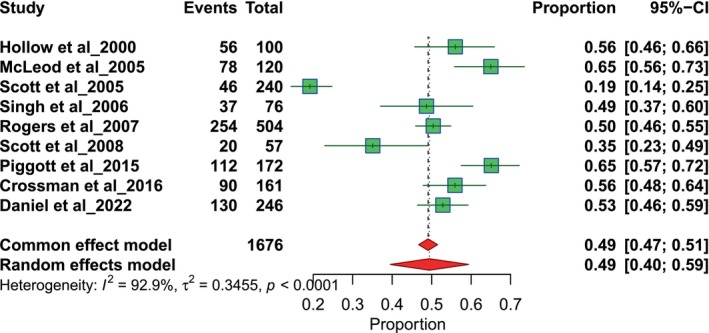
Overall pooled GP referral.

**FIGURE 3:**
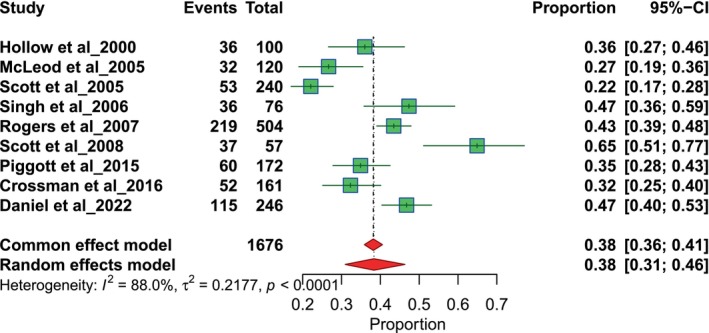
Overall pooled Dentist referral.

The results of nine studies focused on general practitioners (GP). The referral proportions range from 0.19 to 0.65. The pooled proportion for GPs is 0.49, with a 95% CI of 0.40 to 0.59, derived using a random‐effects model (Figure 2). The results of nine studies assessed referral proportions related to dentists. Each study reports the number of events and total participants, with proportions ranging from 0.22 to 0.65. The pooled proportion for dentists is 0.38 with a 95% confidence interval (CI) of 0.31 to 0.46 using a random‐effects model (Figure 3).

### 3.6 The Relative Risk of GP and Dentist Referrals

**FIGURE 4:**
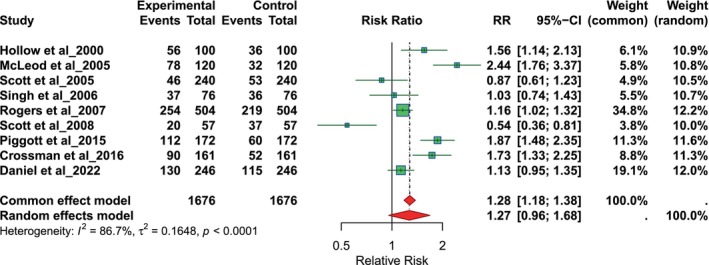
Overall pooled relative risk (RR) of GP and dentist referrals.

The pooled relative risk of referrals made by GPs compared to dentists was 1.27 (95% CI: 0.96–1.68) using a random‐effects model, indicating no statistically significant difference since the confidence interval includes 1.0 (Figure 4). Substantial heterogeneity was observed (*I* ^2^ = 86.7%, *p* < 0.0001), suggesting considerable variability across studies.

This indicates that GPs referred patients at a 27% higher rate than dentists. The results show that GPs might refer patients more often than dentists, which could be due to inequalities in access to specific dental procedures or differences in the range of care these professionals offer. The observed confidence interval, while relatively narrow, reflects a moderate level of precision in the pooled estimate; thus, the actual relative risk is likely to fall within this range. These results underline the importance of understanding referral patterns to inform healthcare policies to improve patient flow and optimise resource allocation between healthcare providers. Such insights can contribute to ensuring equitable and efficient access to care, addressing potential gaps in service provision, and supporting better coordination between GPs and dentists.

### 3.7 Proportions and Trends in Referral Delays

**FIGURE 5:**
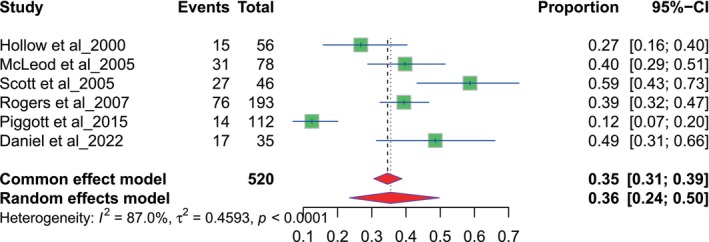
Overall pooled proportion of GP delays.

**FIGURE 6:**
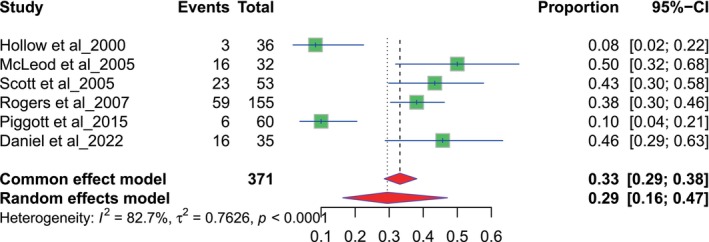
Overall pooled proportion of Dentist delays.

The pooled proportion of GP delays was estimated with six selected studies with a total sample size of 520 patients. The observed pooled proportion for GP delays was [0.36 (36%), random effects model], with a 95% confidence interval (CI) of [0.24–0.50] (Figure 5). Six studies were used to study dentist delays, involving a total sample of 371 individuals. The pooled proportion of delays was [0.29 (29%), random effects model], with a 95% CI of [0.16–0.47] (Figure 6). On average, GP delays were slightly higher than dentist delays, as reflected in the pooled proportions. Both analyses demonstrated high heterogeneity, underscoring the need for caution in interpreting pooled estimates. The variability in delays may reflect differences in access and referral practices. The findings suggest that GP referrals experience slightly higher delays than dentist referrals. This highlights the need for improved awareness among healthcare providers about the urgency of timely referrals to minimise delays in diagnosis and treatment.

### 3.8 Delay Time (Weeks) of GP and Dentist

The median delay for both GPs and dentists is approximately 12.99 weeks, indicating that half of the cases are resolved within this timeframe. The mean delay, however, slightly differs between the two groups, with GPs exhibiting a mean delay of 12.32 weeks compared to 11.32 weeks for dentists. These findings suggest that, on average, the referral timelines for GPs and dentists are similar, with marginally longer delays for GPs. The variability in referral delays, measured by standard deviation, is higher for GPs (10.33 weeks) than for dentists (8.95 weeks). This indicates a greater inconsistency in referral times for GPs.

Additionally, delays range from 2 to 25 weeks for GPs and 2 to 20 weeks for dentists, highlighting the wider spread among GPs. This variability suggests that procedural or systemic inefficiencies might contribute to GP referrals. Extreme delays, defined as referrals taking more than 21 weeks, were observed in 16.67% of GP cases (1 out of 5).

In contrast, no such extreme delays were noted among dentists, suggesting that dentists are less likely to exhibit outliers. The absence of extreme delays for dentists may reflect a more controlled workflow, even if some delays occur due to clinical interventions. While GPs and dentists show comparable median and mean delays, GPs exhibit greater variability and are solely responsible for the extreme delays observed. These findings imply that systemic inefficiencies may disproportionately affect GP referrals or that specific cases managed by GPs are more prone to longer delays. On the other hand, dentists may maintain a more consistent referral process, potentially due to the structured nature of their clinical interventions.

### 3.9 Referral Stage by GP and Dentist

Meta‐analysis could not be carried out since only one study reported on the disease stage at the time of referral. It was reported that there was no statistically significant difference in the disease stage at diagnosis between referrals from a dentist and a GP (odds ratio [OR] 1.85, 95% CI = 0.8 to 4.1). The study reported 41.3% (*n* = 19) Stage I/II (early stage) for GP, while for the same stage, it was 56.6% (*n* = 30) for Dentist. Regarding late stage (Stage III/IV), GP was 58.7% (*n* = 27) and 43.3% (*n* = 23) for Dentists.

### 3.10 Publication Bias and Sensitivity Analysis

**FIGURE 7:**
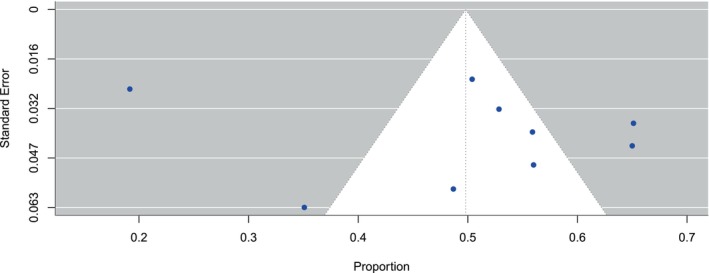
Funnel plot for publication bias.

**FIGURE 8:**
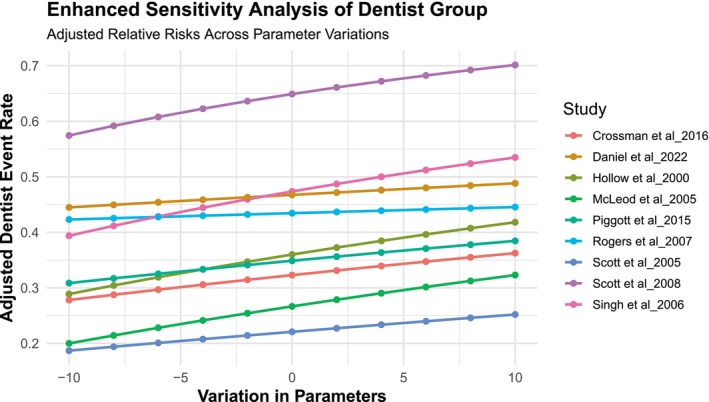
Sensitivity analysis of Dentist.

**FIGURE 9:**
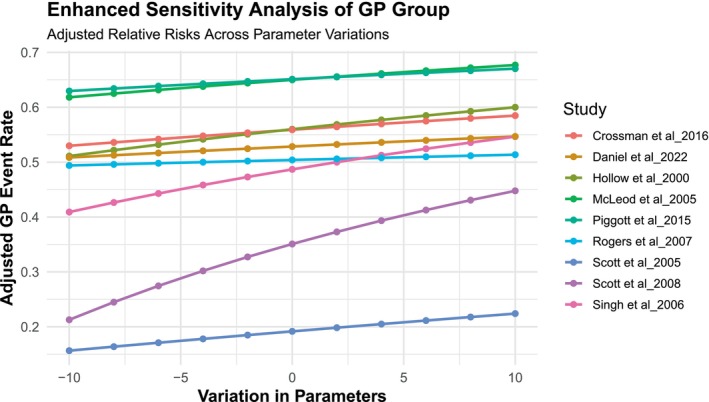
Sensitivity analysis of GP.

Egger's test and Begg's funnel plot were utilised to evaluate the publication bias of the studies included in our study. The symmetrical funnel plot for the various outcomes indicated that the results of our analysis were not affected by publication bias (Figure 7). Egger's test results indicated no bias in our selected studies, as the *p*‐values for all outcomes exceeded 0.05 (*p* = 0.8088). We employed sensitivity analysis to assess the stability of our results (Figures 8 and 9). Sensitivity analysis was conducted to evaluate the robustness of our research findings. The sensitivity curves for most studies were flat or only slightly sloped, indicating consistent outcomes. The statistical significance of the outcomes remained unchanged upon the exclusion of any specific article, affirming the legitimacy and stability of our research findings and showing that the estimations are highly reliable and robust to parameter changes.

### 3.11 Risk of Bias Assessment

**FIGURE 10:**
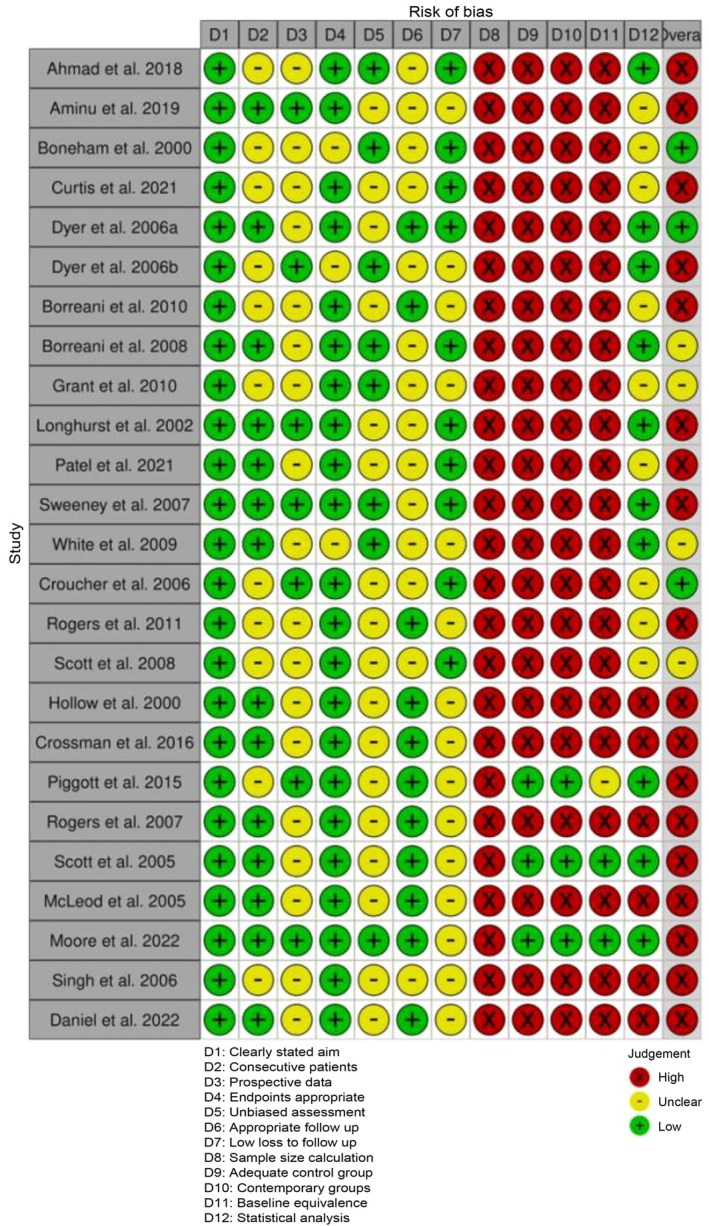
Risk of Bias assessments among studies.

**FIGURE 11:**
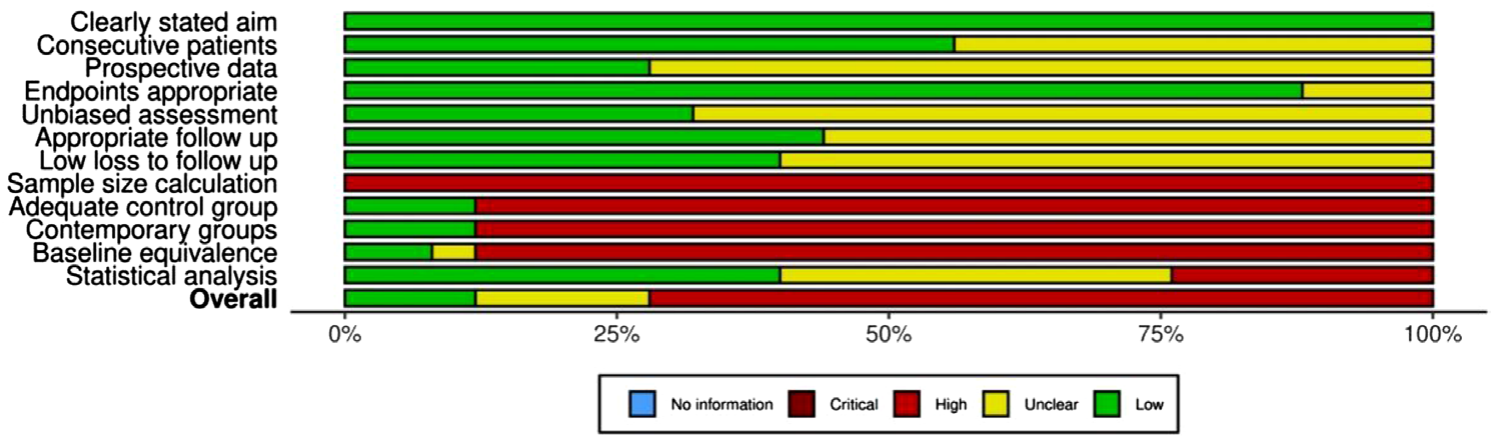
Summary plot for the selected studies.

Risk of bias was assessed across all 25 included studies using the MINORS criteria. As summarised in Figure 10, 18 studies were judged to have a high overall risk of bias, 4 studies showed a moderate risk, and 3 studies were classified as low risk. Common areas of high risk included unbiased assessment of endpoints, adequate control groups, contemporary comparisons, and statistical analysis. The risk of bias assessments, conducted using the MINORS tool, are visualised in Figures 10 and 11.

## 4 Discussion

This study explored the multifaceted barriers to early diagnosis of oral cancer in the UK, focusing on patient behaviours, healthcare provider practices, and systemic challenges. The analysis affirms that both dentists and general practitioners (GPs) are crucial in referring oral cancer cases for specialised treatment. Our findings revealed that approximately 27 oral malignancies were referred by GPs for every 10 by dentists, though considerable variation existed across the studies. This heterogeneity likely reflects differences in healthcare systems, patient preferences, and accessibility of services. Although the pooled relative risk suggests that GPs may refer more frequently than dentists, this difference was not statistically significant because the confidence interval includes 1.0.

Patient choices regarding which healthcare provider to consult first are influenced by several factors, including the location of symptoms, perceived accessibility, and socioeconomic status. Research indicates that dentists are more likely to refer cases involving the dentition or denture‐bearing areas, while patients with general oral discomfort or broader health concerns are more likely to consult GPs (Daniel and Rogers 2022; Langton et al. 2020; Varela‐Centelles et al. 2017, 2021). Socioeconomically disadvantaged individuals also tend to present more frequently to GPs for oral issues, likely due to better access and the perception of lower costs associated with medical consultations (Cope, Chestnutt, et al. 2016; Cope, Francis, et al. 2016; Perera et al. 2023; Ravaghi et al. 2020; Teoh et al. 2023).

Dental access challenges remain a significant issue in the UK. According to NHS statistics, nearly 60% of adults had not visited an NHS dentist in the previous year (NHS 2024), with access levels falling below pre‐pandemic rates. Initiatives such as the £20,000 ‘golden hello’ scheme to attract dentists to underserved areas and plans for mobile dental vans have faced delays, limiting their intended impact on service accessibility (NHS 2024). These gaps underscore persistent systemic barriers that hinder early oral cancer detection.

Our findings also indicate differences in the stage of cancer presentation between referrals from dentists and GPs. Although no statistical difference was observed, there was a trend towards earlier‐stage diagnoses from dental referrals. This may be due to dentists detecting asymptomatic lesions during routine care, an advantage less common in GP practice, where oral cancer cases are relatively rare. For instance, Scott et al. (2005) reported that 15% of dental referrals involved asymptomatic findings, compared to just 1.4% from GPs (Scott et al. 2005).

Healthcare professional knowledge gaps may further contribute to delays. Although dentists are more likely to perform routine oral examinations, some studies indicate that GPs also frequently examine the oral cavity when patients report discomfort (González‐Moles et al. 2022; Macpherson et al. 2003). However, barriers such as time constraints and limited training remain. Interestingly, some researchers argue that GPs may be better positioned for opportunistic screening, given their regular contact with patients with comorbidities (Carter and Ogden 2007; Holmes et al. 2003).

Despite the potential benefits of screening, no formal oral cancer screening programme exists in the UK (Speight et al. 2017). Opportunistic screening has been proposed for high‐risk groups, but infrequent dental attendance limits its feasibility. Patients should be encouraged to attend routine dental appointments, where asymptomatic lesions are more likely to be detected. Furthermore, thorough patient histories and vigilant clinical assessment during medical consultations remain critical, especially in the absence of obvious lesions.

The analysis of diagnostic delays highlighted inconsistent reporting across studies, complicating quantitative synthesis. Definitions of ‘professional delay’ varied, with some studies considering delays beyond 6 days, and others setting the threshold at 2 weeks (McLeod et al. 2005; Onizawa et al. 2003). Although no substantial difference between GP and dental referrals was evident, there was a trend towards slightly longer delays in GP‐initiated cases. Updated NICE guidelines recommend that patients with suspicious oral lesions be referred within 2 weeks of GP consultation (NICE 2018). However, unclear referral pathways and patient factors continue to hinder prompt referrals (Grimes et al. 2017).

Data from the National Cancer Diagnosis Audit shows that approximately 60% of oral cancer cases were referred by GPs using urgent referral pathways, but dental referrals were underrepresented in this audit (Swann et al. 2018). Diagnostic pathways remain complex, with some diagnoses achieved at the primary care level through biopsy, yet many primary care dentists lack experience in performing biopsies (Diamanti et al. 2002; Schiavo‐Di Flaviano et al. 2025). This hesitancy, often linked to medico‐legal concerns and limited exposure to cancer cases, highlights the importance of prompt referrals to secondary care and the production of high‐quality referral letters (Björkeborn et al. 2017; Varela‐Centelles et al. 2017).

Addressing these challenges requires greater integration between medical and dental services and clearer, auditable referral pathways. NICE has proposed free dental access for patients with suspected oral cancer, but implementation remains unclear (NICE 2018). Further research is needed to evaluate the impact of such policies and to establish best practices for early detection across primary care settings.

The findings of this review highlight the urgent need for multifaceted interventions to promote the early diagnosis of oral cancer. Public health campaigns should focus on raising awareness of oral cancer symptoms, reducing stigma, and encouraging regular dental attendance, particularly in underserved populations. Enhancing the training of general practitioners and dentists in the recognition of early oral malignancies is crucial, supported by continued professional development programmes. Furthermore, improving integration between medical and dental services, establishing clear and auditable referral pathways, and addressing workforce shortages could significantly reduce diagnostic delays. Policymakers should prioritise the implementation of initiatives aimed at improving accessibility to NHS dental services, while future research should explore the effectiveness of integrated care models and strategies to strengthen communication between healthcare providers. Collectively, these measures could facilitate earlier detection, expedite treatment initiation, and improve outcomes for patients with oral cancer.

While this systematic review and meta‐analysis offer valuable insights into oral cancer diagnostic delays and health‐seeking behaviours in the UK, several limitations must be acknowledged. One major limitation is the potential for publication bias, which may result in the underreporting of studies with negative or null findings and, consequently, an overestimation of the impact of certain interventions or risk factors. Additionally, many of the included studies relied on patient self‐reported data, which is susceptible to social desirability and recall biases, especially when addressing sensitive issues such as stigma, fear, and delays in seeking care. For example, patients may underreport their hesitancy to seek medical attention due to fear or embarrassment, potentially affecting the accuracy of reported barriers. The diversity of study designs and methodologies across the included papers also presented challenges. Despite efforts to standardise data extraction, variations in study settings, sample sizes, and outcome measures may have introduced inconsistencies and affected the generalisability of the findings. Finally, although the review was restricted to English‐language publications, which may have excluded some relevant studies, this decision was based on resource limitations, concerns about translation accuracy, and the predominance of English‐language research within UK healthcare literature. We believe this restriction had minimal impact on the overall conclusions of the review.

## 5 Conclusion

In conclusion, this study provides important insights into health‐seeking behaviours and diagnostic delays related to oral cancer in the UK. Patient‐level barriers, including fear, stigma, and lack of awareness, alongside system‐level challenges such as referral delays and poor coordination between primary and secondary care, were found to contribute to late diagnoses. Addressing these barriers through public health campaigns, enhanced training for healthcare practitioners, and integrated referral pathways is essential to promote earlier detection. Timely diagnosis is critical to improving prognosis and survival rates in oral cancer. Further research should explore psychosocial factors, healthcare accessibility, and policy interventions to strengthen early detection strategies and improve patient outcomes.

## Author Contributions

**Emmanuel Kwateng Drokow:** conceptualization, methodology, software, investigation, writing – original draft, writing – review and editing, formal analysis. **Michael Drinnan:** conceptualization, methodology, supervision, writing – review and editing. **Cecilia Amponsem‐Boateng:** software, methodology, writing – review and editing, formal analysis, validation. **Fatemeh Vida Zohoori:** supervision, writing – review and editing, conceptualization. **Janet A. Wilson:** supervision, writing – review and editing, conceptualization. **Kamini Shah:** supervision, writing – review and editing, conceptualization. **Francisca Arboh:** writing – review and editing, methodology, software, validation.

## Ethics Statement

The authors have nothing to report.

## Consent

The authors have nothing to report.

## Conflicts of Interest

The authors declare no conflicts of interest.

## Supporting information


**Table S1:** Search strategies were tailored to retrieve only UK‐based studies by using geographic terms (e.g., “United Kingdom,” “UK,” etc.) and limiting to English‐language publications from 2000 to 2024. Each database query combined four concept categories—Condition (oral cancer), Delay Type (diagnostic delay), Health‐Seeking Behaviour and Geography (UK) – using Boolean logic and truncation. The full search strings and field syntax for PubMed, Scopus, Web of Science, EMBASE and Google Scholar are detailed below, in accordance with PRISMA‐S reporting recommendations.

## Data Availability

The data that support the findings of this study are available on request from the corresponding author. The data are not publicly available due to privacy or ethical restrictions.
