# Supplementary material for: Patient and System Barriers to Early Diagnosis of Oral Cancer in the UK
Source: Oral Dis. 2025 Nov 15;32(3):688–701. doi: 10.1111/odi.70125 (PMC13125732; doi:10.1111/odi.70125)
Supplement: Supplementary file 1 — Table S1: Search strategies were tailored to retrieve only UK‐based studies by using geographic terms (e.g., “United Kingdom,” “UK,” etc.) and limiting to English‐language publications from 2000 to 2024. Each database query combined four concept categories—Condition (oral cancer), Delay Type (diagnostic delay), Health‐Seeking Behaviour and Geography (UK) – using Boolean logic and truncation. The full search strings and field syntax for PubMed, Scopus, Web of Science, EMBASE and Google Scholar are detailed below, in accordance with PRISMA‐S reporting recommendations. [file ODI-32-688-s001.docx]

Search strategies were tailored to retrieve only UK-based studies by using geographic terms (e.g. “United Kingdom,” “UK,” etc.) and limiting to English-language publications from 2000–2024. Each database query combined four concept categories – **Condition** (oral cancer), **Delay Type** (diagnostic delay), **Health-Seeking Behaviour**, and **Geography** (UK) – using Boolean logic and truncation. The full search strings and field syntax for PubMed, Scopus, Web of Science, EMBASE, and Google Scholar are detailed below, in accordance with PRISMA-S reporting recommendations.

Supplementary Table 1.

| Database | Concept Category | Search Terms | Boolean Operators / Truncation Used | Database-Specific Notes/Syntax |
| --- | --- | --- | --- | --- |
| **PubMed** | Condition (oral cancer) | MeSH: “Mouth Neoplasms”[Mesh] <br> Free-text: (mouth OR oral OR tongue OR lip OR palate OR buccal OR gingiva) AND (cancer OR carcinoma *OR neoplasm*) | OR, AND; * for truncation; quotes for phrases | Use [Mesh] for controlled terms and [Title/Abstract] for free text; limit to English, dates 2000–2024. |
|  | Delay Type | MeSH: “Delayed Diagnosis”[Mesh] <br> Free-text: (“delayed diagnosis” OR “diagnostic delay” OR patient delay OR “late diagnosis”) | OR, AND; quotes for phrases | Fields [Mesh] and [Title/Abstract] used; combine with Condition terms via AND. |
|  | Health-Seeking Behaviour | Free-text: (“health-seeking” OR “help-seeking” OR “care-seeking” OR “patient behavior” OR “patient behaviour”) | OR; quotes for phrases | Use Title/Abstract fields; synonyms may be hyphenated; AND with other concepts. |
|  | Geography (UK) | MeSH: “United Kingdom”[Mesh] <br> Free-text: (“United Kingdom” OR UK OR England OR Scotland OR Wales OR “Northern Ireland”) | OR; quotes for multi-word terms | No built-in country filter in PubMed; use terms in title/abstract; AND with other concepts. |
| **Scopus** | Condition (oral cancer) | TITLE-ABS-KEY: (mouth OR oral OR tongue OR lip OR palate OR buccal OR gingiva) AND (cancer OR carcinoma *OR neoplasm*) | OR, AND; * for truncation | Search fields TITLE-ABS-KEY; apply year (2000–2024) and English filters; country filter = UK. |
|  | Delay Type | TITLE-ABS-KEY: (“delayed diagnosis” OR “diagnostic delay” OR patient delay OR “late diagnosis”) | OR; quotes for phrases | As above; combine with Condition terms via AND. |
|  | Health-Seeking Behaviour | TITLE-ABS-KEY: (“health-seeking” OR “help-seeking” OR “care-seeking” OR “patient behavior” OR “patient behaviour”) | OR; quotes for phrases | As above. |
|  | Geography (UK) | TITLE-ABS-KEY: (“United Kingdom” OR UK OR England OR Scotland OR Wales OR “Northern Ireland”) | OR; quotes for multi-word terms | Use Scopus country/region filter to limit to UK; combine via AND with other terms. |
| **Web of Science** | Condition (oral cancer) | TS=(mouth OR oral OR tongue OR lip OR palate OR buccal OR gingiva) AND (cancer OR carcinoma *OR neoplasm*) | OR, AND; * for truncation | Use TS= (Topic) search; refine results by “Countries/Regions: United Kingdom”; limit to 2000–2024, English. |
|  | Delay Type | TS=(“delayed diagnosis” OR “diagnostic delay” OR patient delay OR “late diagnosis”) | OR; quotes for phrases | As above; AND with other concepts. |
|  | Health-Seeking Behaviour | TS=(“health-seeking” OR “help-seeking” OR “care-seeking” OR “patient behavior” OR “patient behaviour”) | OR; quotes for phrases | As above. |
|  | Geography (UK) | TS=(“United Kingdom” OR UK OR England OR Scotland OR Wales OR “Northern Ireland”) | OR; quotes for multi-word terms |  |
| **EMBASE** | Condition (oral cancer) | Emtree: ‘mouth neoplasm’/exp OR ‘oral neoplasm’/exp OR ‘lip neoplasm’/exp<br> Free-text: (mouth neoplasm OR oral neoplasm OR lip neoplasm OR “oral cancer” OR “mouth cancer”) | OR; * for truncation |  |
|  | Delay Type | Emtree: ‘delayed diagnosis’/exp OR ‘late diagnosis’/exp<br> Free-text: (“delayed diagnosis” OR “diagnostic delay” OR patient delay OR “late diagnosis”) | OR; quotes for phrases | As above; AND with condition terms. |
|  | Health-Seeking Behaviour | Free-text: (“health seeking” OR “help seeking” OR “care seeking” OR “patient behavior” OR “patient behaviour”) | OR; quotes as needed | As above. |
|  | Geography (UK) | Emtree: ‘united kingdom’/exp OR ‘england’/exp OR ‘scotland’/exp OR ‘wales’/exp OR ‘northern ireland’/exp<br> Free-text: (“United Kingdom” OR UK OR England OR Scotland OR Wales OR “Northern Ireland”) | OR; quotes for multi-word terms |  |
| **Google Scholar** | Condition (oral cancer) | Free-text: “oral cancer” OR “mouth cancer” OR “oral neoplasm” | OR; quotes for exact phrases | No controlled vocabulary; use quotes for phrases; restrict by date and language (UI filters). |
|  | Delay Type | Free-text: “delayed diagnosis” OR “diagnostic delay” OR “late diagnosis” OR “patient delay” | OR; quotes for phrases | As above. |
|  | Health-Seeking Behaviour | Free-text: “health-seeking behavior” OR “health-seeking behaviour” OR “help-seeking” OR “patient behavior” | OR; quotes for phrases | As above. |
|  | Geography (UK) | Free-text: “United Kingdom” OR UK OR “Great Britain” OR England OR Scotland OR Wales OR “Northern Ireland” | OR; quotes for multi-word terms | As above; include “United Kingdom” or related terms in search string. |

All searches were limited to English-language records and publication dates Jan 2000–Dec 2024. Wildcard/truncation (*) captures term variants (e.g. carcinoma/carconomas), and all concept categories were combined with AND in each database query. The above strategies ensure inclusion of UK-relevant studies only.
